# Protocol for inducing hippocampal formation lesions and associated behavioral testing in Japanese quail

**DOI:** 10.1016/j.xpro.2022.101553

**Published:** 2022-07-18

**Authors:** Chelsey C. Damphousse, Noam Miller, Diano F. Marrone

**Affiliations:** 1 Wilfrid Laurier University, Waterloo, ON N2L 3C5, Canada

**Keywords:** Model Organisms, Neuroscience, Behavior

## Abstract

Here, we present a protocol for inducing selective lesions in the hippocampal formation of Japanese quail (*Coturnix japonica)*, coupled with associated behavioral testing. We first describe the surgical procedure for aspiration lesions in Japanese quail. We then detail two well-known hippocampus-dependent behavioral tests adapted to birds—foraging array (FA) and spontaneous object recognition (SOR). This protocol is adapted from those used in mammals and can be used to study the involvement of Japanese quail memory centers in declarative memory.

For complete details on the use and execution of this protocol, please refer to Damphousse et al. (2022).

## Highlights

• Protocol for characterizing brain function in Japanese quail (*Coturnix japonica*)
• Surgical procedure to create craniotomies and aspiration lesions
• Instruction for two memory tasks: foraging array and spontaneous object recognition
• Can be easily adapted to analysis of other avian species or brain regions

## Abstract (editor highlights)

Publisher’s note: Undertaking any experimental protocol requires adherence to local institutional guidelines for laboratory safety and ethics.

## Graphical abstract

**Figure:**
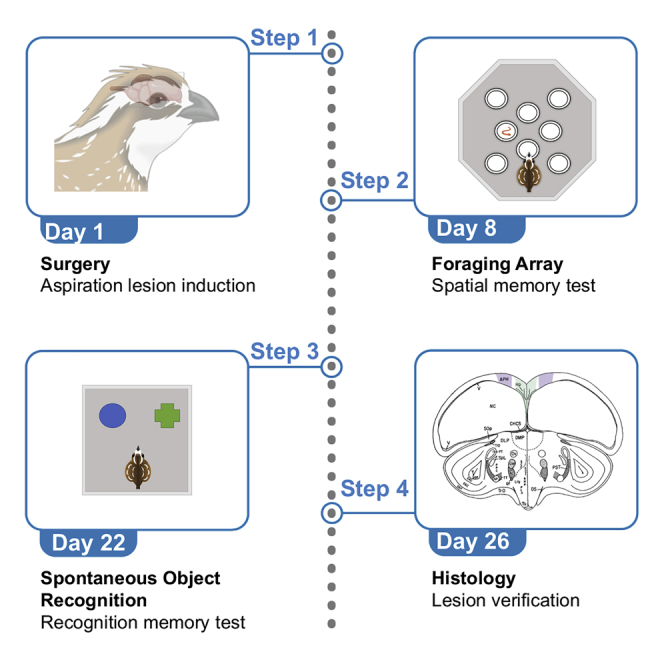

## Before you begin

The protocol below describes aspiration lesions in Japanese quail. Behavioral testing outlined is specific to the hippocampus (Hp) and area parahippocampalis (APH) as described in the study by Damphousse and colleagues (2022). This protocol can be adapted for multiple brain regions and/or avian species, provided the appropriate brain atlas, or experience, to establish coordinates.

In the described behavioral testing, FA procedures followed methods previously described by Lormant et al. (2018) and SOR protocol was adapted from a previous publication testing Japanese quail (Damphousse et al., 2021). These procedures were developed with female quail, but the authors foresee no issues with utilizing the procedures as described with males. The authors also have experience completing these procedures in a wide range of ages of quail without the need to change any of the procedures described here.

Note also that, based on observed effect sizes, six quail should be considered the minimum number to include in each lesion group. With any lesion experiment, there is some risk of losing animals due to either mortality or the development of post-surgical behavioral abnormalities (commonly lethargy) that prevent behavioral testing, so it may be advisable to lesion more than this minimum number.

### Institutional permissions

Procedures used here were approved by the Animal Care Committee (ACC) of Wilfrid Laurier University in accordance with the guidelines of the Canadian Council on Animal Care. Experimenters were trained in Japanese quail handling procedures and underwent surgical training by ACC staff before performing the following experiments.

Before carrying out the following, be sure to acquire permissions from your institution.

## Key resources table

**Table:** 

REAGENT or RESOURCE	SOURCE	IDENTIFIER
**Chemicals, peptides, and recombinant proteins**		

lidocaine and epinephrine (2%, pre-mixed 1:1, 1 mL)	Bimeda, Cambridge, ON	1LID010
Opthalmic gel	CDMV	N/A
antibacterial cleanser (Phenrex)	CDMV	N/A
chlorhexidine gluconate solution (Baxedin)	CDMV	N/A
Isopropyl alcohol	Sigma-Aldrich, Oakville, ON	I9030-4L
Isoflurane	CDMV	N/A
Enrofloxacin (Baytril)	CDMV	N/A
Ketoprofen (Anafen)	CDMV	N/A
2-methylbutane	Sigma-Aldrich, Oakville, ON	270342
Nuclear fast red	Sigma-Aldrich, Oakville, ON	60700

**Experimental models: Organisms/strains**		

Japanese Quail (*Coturnix japonica*) female, approx. 3 months of age	Spring Creek Quail Farm, Saint Anns, ON	N/A

**Software and algorithms**		

ANY-maze	Stoelting	4.0 www.any-maze.com
JASP	JASP team, 2021	0.16 www.jasp-stats.org

**Other**		

Cryostat	Leica	CM3050
Brightfield microscope	Olympus	BX53M
SomnoSuite™ anesthesia system	Kent Scientific, Torrington, CT	N/A
RightTemp™ warming pad and reulator	Kent Scientific	N/A
Quail (*Coturnix japonica*) brain atlas	Baylé et al., 1974	N/A
Stereotaxic apparatus	Kopf Instruments, Tujunga, CA	Model 963
Dry glass bead sterilizer	Cole-Parmer Canada Company, Montreal, QC	RK-10779-10
Embedding medium	VWR	95057-838
Superfrost Plus™ slides	VWR	95057-985
Syringe (1 mL)	VWR	CABD309659
Needles (25 gauge × 5/8″)	VWR	CA76313-818
Cotton gauze	VWR	CA95038-722
Cotton swabs	VWR	CA89133-752
Sterile saline	VWR	CA101320-568
Hemostats	World Precision Instruments (WPI)	555223F
Forceps	WPI	WP4000
needle drivers	WPI	14110
Spatula	WPI	504022
Scalpel	WPI	500236-G
Scalpel blades	WPI	504169
Bone wax	Fisher Scientific	501180260
Silk sutures	Stoelting	10000657
Hemostatic sponges	DSI Dental Solutions Israel	DSP-32
Cordless rotary tool	Dremel	7760
Engraving bit	Dremel	106
Pasteur pipette	VWR	14672-400
Pipette bulb	Fisherbrand	13-681-51
Plastic sealing wrap (Glad® Press’n Seal or similar)	Amazon	N/A
Calipers	Amazon	N/A
3.25 oz. Disposable Portion Cups with Lids	Amazon	N/A
2 oz. Disposable Portion Cups with Lids	Amazon	N/A
White corrugated plastic sheeting	The Home depot	1000171722

## Materials and equipment

***Alternatives:*** This protocol uses a pipette bulb for manual aspiration of brain tissue. The aspirations could also be completed with a vacuum line, either connected to a central line or vacuum pump. In this case, a vessel should be placed between the source of the vacuum and the pipette tip to contain the aspirated tissue. Note that while aspiration lesions are well suited to regions at or near to brain’s surface (as is the case here), chemical or electrolytic lesions may be more appropriate for lesions of deeper brain structures.

Materials for all surgeries:

• Enrofloxacin.
• Ketoprofen.
• Lidocaine.
• Isoflurane anesthesia system.
• Surgical skin disinfectant: antibacterial cleanser, alcohol and baxedin.
• Ophthalmic gel.
• Scalpel handle and blade.
• Dental spatula.
• 1 mL syringes.
• Sterile saline.
• Fine forceps.
• Hemostats.
• Needle Driver.
• Rotary Tool.
• Calipers.
• Drill bit – 1 mm tip.
• Pasteur Pipette.
• Pipette bulb.
• Bone wax.
• Hemostatic sponge.
• Stereotax (Kopf).
• Sutures.
• Heating pad.

## Step-by-step method details

### Surgical procedure for aspiration lesions in Japanese quail

**Timing: 1 h per subject**

This step describes how to create craniotomies and aspiration lesions in Japanese quail, as well as manage pre-surgical and post-surgical care.

***Note:*** Sham lesions can be created by following all step outlined below with the exclusion of aspirations outlined in step 11.

1. Before starting surgery, determine target coordinates for aspiration lesion. Using the atlas described by Baylé and colleagues (1974), Hp lesions should include the area spanning 5 mm anterior to bregma, 3 mm posterior, 1.5 mm on either side of interfrontal suture, and 3 mm in depth. APH lesions are 5 mm anterior to bregma, 3 mm posterior, 1.5 mm–3.5 mm lateral to bregma, 2 mm deep.

**CRITICAL:** It is essential for surgery to be carried out in a sterile environment. Surgical instruments must be autoclaved prior to surgery day and sterilized between subjects (if more than one surgery is occurring per surgery day). Decontamination with 75% alcohol may be used in addition to bead sterilization.

***Note:*** Quail are not fasted prior to surgery here. However, 1 h of fasting prior to avian surgery is common as it is thought that an empty crop and stomach can reduce the risk of regurgitation and subsequent aspiration on gastric contents (Curro, 1998; but see Jenkins, 2000).

***Note:*** Coordinates given are for Japanese quail and were determined using the atlas described by Baylé and colleagues (1974). This protocol can readily be adapted for use with other birds by utilizing an atlas appropriate for the desired species.

2. Prepare subject for surgery.
  a. Weigh quail and administer analgesic (5 mg/kg) and antibiotic (5 mg/kg) subcutaneously. Record quail weight, the rate and concentration of drug, and dose administered into surgical log (see Data S1 for example).
    ***Note:*** Calculate the volume of drug to administer by multiplying the weight of the subject (kg) by the rate of the drug (mg/kg) and then dividing by the drug concentration (mL/mg).
    ***Note:*** A relatively straightforward injection location can be found under the wing against the body of the subject. Here you should find an area with fewer feathers where the skin will be visible.
  b. Sedate quail using gradual fill isoflurane in induction chamber 5% for induction delivered in 500 mL/min oxygen.
    ***Note:*** While not necessary, covering the induction chamber with a shroud can be helpful in minimizing stress to the subject.

3. When righting reflex has ceased, move subject to stereotax, prone position, secure ear bars, secure beak into nosecone.

**CRITICAL:** Use a heating pad to maintain body temperature during surgery, aiming for 35°C–40°C. Be sure that this temperature is not exceeded as overheating can lead to mortalities.

**CRITICAL:** Ear bar insertion should be done with extreme caution. If they are too loose, the head will not be secure during the procedure. Too tight, and there is a risk of rupturing the subject’s eardrum. Ensure ear bars are inserted evenly on both sides. The average ear bar insertion for Japanese quail in the procedure described here was 1.2cm.

**CRITICAL:** After securing ear bars and nosecone, ensure that the head is unable to move and will remain stationary during the following steps. Skull movement during craniotomy and aspiration will cause inaccuracies when locating the target.

***Note:*** An internal probe may be used to monitor body temperature, however, setting your heating device to an output within the range described is also sufficient.

***Note:*** Several methods are available for securing the beak within a nosecone. One method is to use a rat nose cone. For quail, secure the head by closing the beak, placing the bite bar under the beak, and gently adjusting the nose cone so that the top of the bill is secured against the internal rubber lining of the nose cone. This method was utilized in the study by Damphousse et al. (2022) and provided consistent lesion placement. An alternative method, placing the bite bar into the gaping beak is outlined in Prather (2012).

4. Continue nosecone anesthesia with isoflurane, 5% for induction, 2%–3% for maintenance delivered in 225 mL/min oxygen.

**CRITICAL:** Monitor anesthesia levels closely throughout the procedure. If the subject is moving or their eyes are open, increase the concentration of isoflurane. In this scenario, adjust isoflurane and restrain the animal to avoid injury until appropriate level of sedation returns. If the subject’s breathing rate has decreased dramatically and their feet appear more blue than red (indicative of blood flow), reduce isoflurane immediately.

5. Pluck feathers from surgery area using forceps.
6. Inject lidocaine subcutaneously at surgical site (1 mL of 2% solution).
7. Administer ophthalmic ointment in both eyes of the animal to protect the corneas from drying and abrasion.

**CRITICAL:** Ophthalmic ointment should be reapplied throughout surgery.

8. Cover the body of the subject with surgical shroud so that only the head and surgical site are uncovered.

***Note:*** Clear plastic sealing wrap (like Glad® Press’n Seal) can be used for this step as it allows for easy monitoring of foot color and respiration, and aids in minimizing heat loss.

9. Prepare the surgical area using antibacterial cleanser, isopropyl alcohol, and chlorhexidine gluconate solution. Repeat this step twice.

**CRITICAL:** Before proceeding with incision, firmly pinch toe to ensure no reflex. If withdrawal reflex is present, wait or increase isoflurane flow.

10. Make an incision over midline of scalp to expose skull. Secure skin using hemostats.
11. Prepare for craniotomy.
  a. Clear the exposed skull of fluid using cotton swabs.
  b. Locate bregma and interfrontal suture (see Figure 1).
    ***Note:*** The interfrontal suture may be difficult to locate. In Japanese quail, there is a portion of the skull over each hemisphere where the skull is very thin and the underlying tissue is visible. If the interfrontal suture is not visible, measuring the distance between these two locations as an approximation of the interfrontal suture has proven to yield symmetrical lesions.
  c. Refer to coordinates for desired lesion and mark these locations on the skull (distance from the interfrontal suture, distance of most rostral and most caudal locations from Bregma). This should result in a rectangular window out of which to drill the craniotomy.
    ***Note:*** Marking can be done by gently scoring the skull surface using a small rotary tool bit or by using a sterilized unlacquered pencil (Terzic et al., 2010).
    ***Note:*** The following is important to consider for bilateral lesions as described in the study by Damphousse and colleague (2022). Hp will only require one craniotomy since this structure is located at the midline. The Hp of both hemispheres can be accessed this way. APH will require two identical craniotomies, one over each hemisphere.
  d. Drill the craniotomy using the rotary tool.
    **CRITICAL:** To ensure the bit does not advance through the skull and into the underlying tissue, great care needs to be taken. When drilling the craniotomy, elbows of the operator should be planted firmly on the surgical table and the tool should be held with both hands. Rather than drilling one side to completion before moving on to the next, the entire perimeter of the craniotomy should be drilled gradually. This involves continually tracing the parameter with the rotary tool in one motion until the desired portion of skull is loose and can be removed in one piece. Removing the skull in this way minimizes risk of damaging underlying tissue with shards of skull.
  e. Remove portion of skull that has been cut away using forceps and ensure craniotomy is free of bone fragments, this can be done with forceps and/or by clearing with a wash of sterile saline.
12. Prepare the tissue for aspiration. Using a pipette and pipette bulb, aspirate the area to predetermined depth.
  a. Using a permanent marker, mark the desired lesion depth from the tip of the Pasteur pipette (3 mm for Hp, 2 mm for APH). Sterilize pipette in bead sterilizer.
    ***Note:*** While aspirations in the study by Damphousse and colleagues (2022) were done free-hand, if a stereotaxic arm is available, the pipette may be secured to this to ensure lesion placement.
  b. Remove dura from the surface of the brain using a needle. This can be accomplished by very gently passing the needle tip over the tissue, snagging the dura so it can be cleared.
    ***Note:*** Using a needle driver or tweezers to slightly bend the tip of the needle into a hook can be a helpful tool in clearing dura.
  c. Secure the bulb to the pipette and begin aspiration. Since this step is done by hand, elbows should be planted firmly on the work surface to provide stability. Suction is created by releasing air from the bulb and then pinching the appropriate valve to allow suction through the pipette tip. Aspirate the tissue within the craniotomy while reaching the desired depth marked on the pipette.
    ***Note:*** At this step, there may be increased bleeding within the craniotomy. Control bleeding by applying sterilized cotton swabs or hemostatic sponge. Ensure bleeding has stopped before proceeding to the following step.

13. Insert hemostatic sponge into craniotomy, fitting snugly and covering exposed brain.

**Figure 1:**
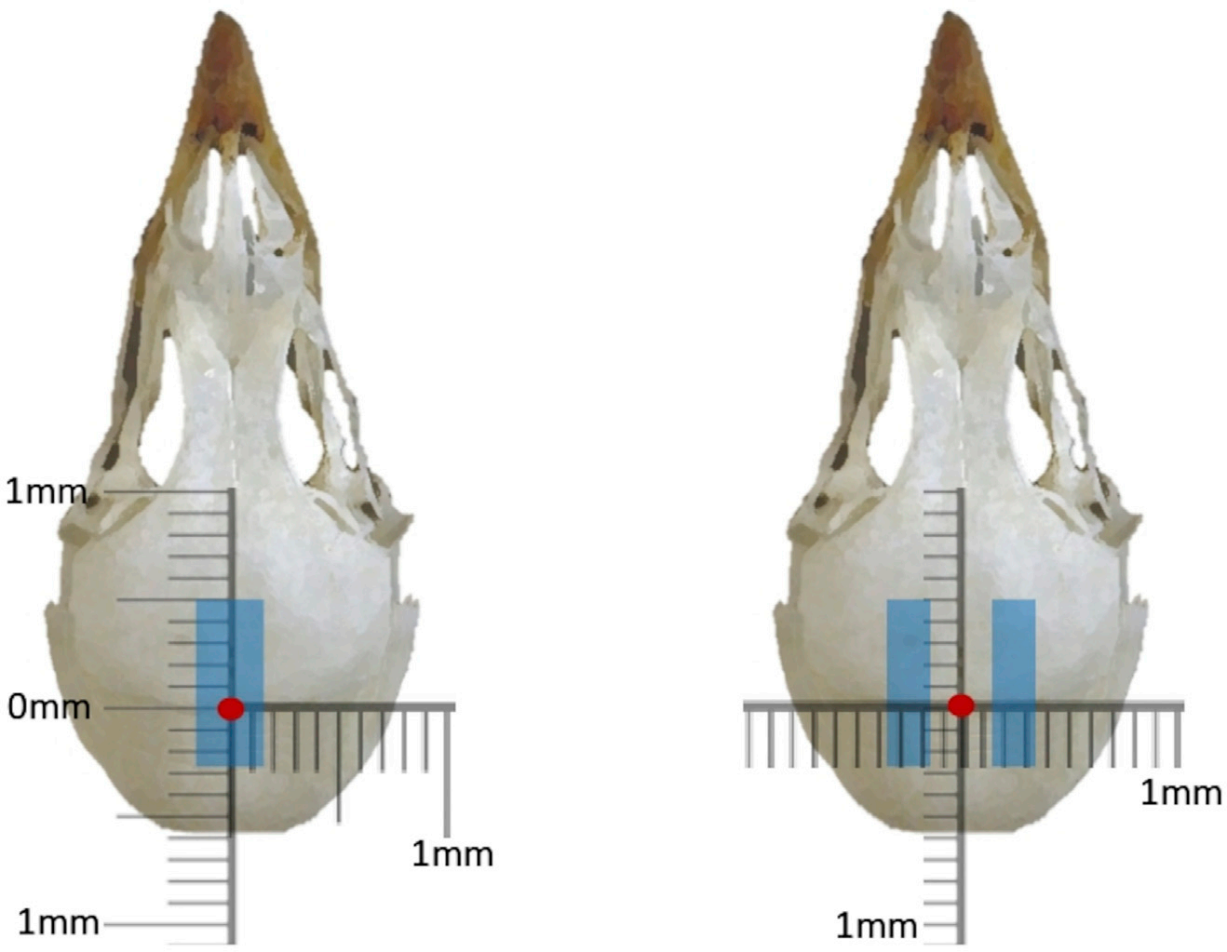
Placement of lesions The left image is a schematic of the Hp lesion and the right, of bilateral APH lesions. The red dot signifies Bregma and the displayed scale is zeroed at this location.
14. Prepare to apply bone wax to seal craniotomy.
  a. Using a scalpel, lightly score the skull around the craniotomy.
  b. Thoroughly dry the scored skull using cotton swabs and clear of any debris.
    ***Note:*** Light scoring helps for better adhesion of the bone wax. If the surface of the skull under the wax is not dry, the bone wax will not adhere.
  c. Heat steel spatula in bead sterilizer or with flame if sterilizer is not available.
  d. Use hot spatula to melt wax over the craniotomy, smoothing the wax and ensuring even distribution over the craniotomy and covering the scored skull.
  e. Allow 2–3 min for the wax to resolidify.
15. Close the incision using an interrupted suture pattern.
16. Remove the ear bars, turn isoflurane flow down to 0% and allow the subject to begin to recover.

**CRITICAL:** Continue heat support as subject is recovering from anesthesia.

17. Once the subject has begun to show movement, swaddle in a clean towel, making sure the wings are secured tightly to the body, and hold until subject is alert and responsive.

**CRITICAL:** During this stage of recovery, quail will often become alarmed and will try to flap their wings while attempting to escape. The subject can cause serious harm to themselves if this occurs so preventative swaddling is necessary.

18. Once the subject’s eyes are open and they can remain upright without assistance, place into recovery cage with heat support and continue to monitor until animal is alert and mobile.

***Note:*** Recovery cage can be covered with a shroud to minimize stress to the animal.

19. Record surgical notes into log (ex. Type of lesion, problems during surgery, number of sutures, etc.).
20. Once recovered, place the subject into individual caging in housing room where they will undergo daily monitoring.

### Post-surgical care

**Timing: 7 days**

The following steps are critical for pain management, condition monitoring, and reducing risk of infection following surgery.

21. Create a log to properly monitor treatment and condition of subjects (see Data S1 for template). This document should include pre-surgical information (as described in the previous section) and post-surgical information described below.
22. Daily monitoring.
  a. Weigh quail and record in log (see Data S1).
    ***Note:*** If weight decreases below a level determined by your veterinarian (usually 70% of the quail’s pre-operative weight), notify the institutional animal care staff and seek potential consultation with your veterinarian (see troubleshooting).
  b. Determine the volume of drug to be administered based on daily weight.
    **CRITICAL:** Treat quail post-surgically with analgesics (e.g., ketoprofen) for 72 h, followed by antibiotic drugs (e.g., enrofloxacin) subcutaneously for up to 1 week.
  c. Record the condition of each subject.
    ***Note:*** Condition can be scored by asking a variety of questions. For example, is the quail bright, alert, and responsive? Does the quail orient toward the door of their cage when the experimenter is present? If no, contact animal care staff as this could indicate that the subject is in pain or needs medical intervention. Record all observations in the post-surgical log.
    **CRITICAL:** Consultation with your institutional veterinarian must be done to establish species specific behaviors associated with pain as well as humane endpoints. Japanese quail specifically should be monitored for anorexia, feather pulling, immobility or problems with stability, and a lack of responsiveness. Again, it is vital to log all observations.
    ***Note:*** If the experimenter is planning on proceeding to the FA procedure, during the 1-week post-surgical care period, supplemental feeding with 3–4 mealworms daily is essential to familiarize quail with the novel food.

### Behavioral experiments

The behavioral tests described in the following section are not mandatory but were used in Damphousse et al. (2022) as one way of assessing the effects of Hp and APH lesions on spatial memory and object recognition memory. If the behavioral protocols are not desired, skip to step 32 for histology protocol to assess lesion placement. Note that training may also take place prior to lesions for some behavioral tasks (particularly tasks that take many trials to train).

### Foraging array

**Timing: 14 days**

The following steps can be used as one way to assess Japanese quail spatial memory.

**Figure 2:**
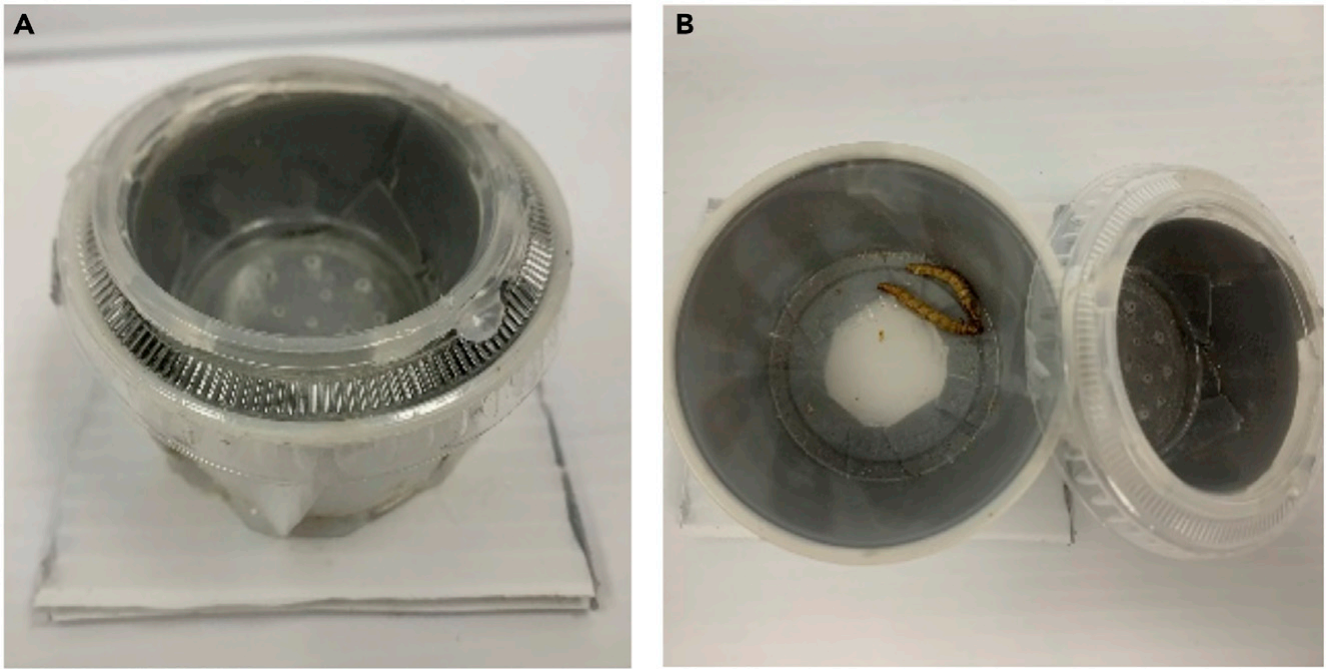
Cups used in FA (A and B) Depicted are (A) a sideview of the cup, as well as (B) a top view of cup with inner perforated cup removed, revealing mealworms.

**Figure 3:**
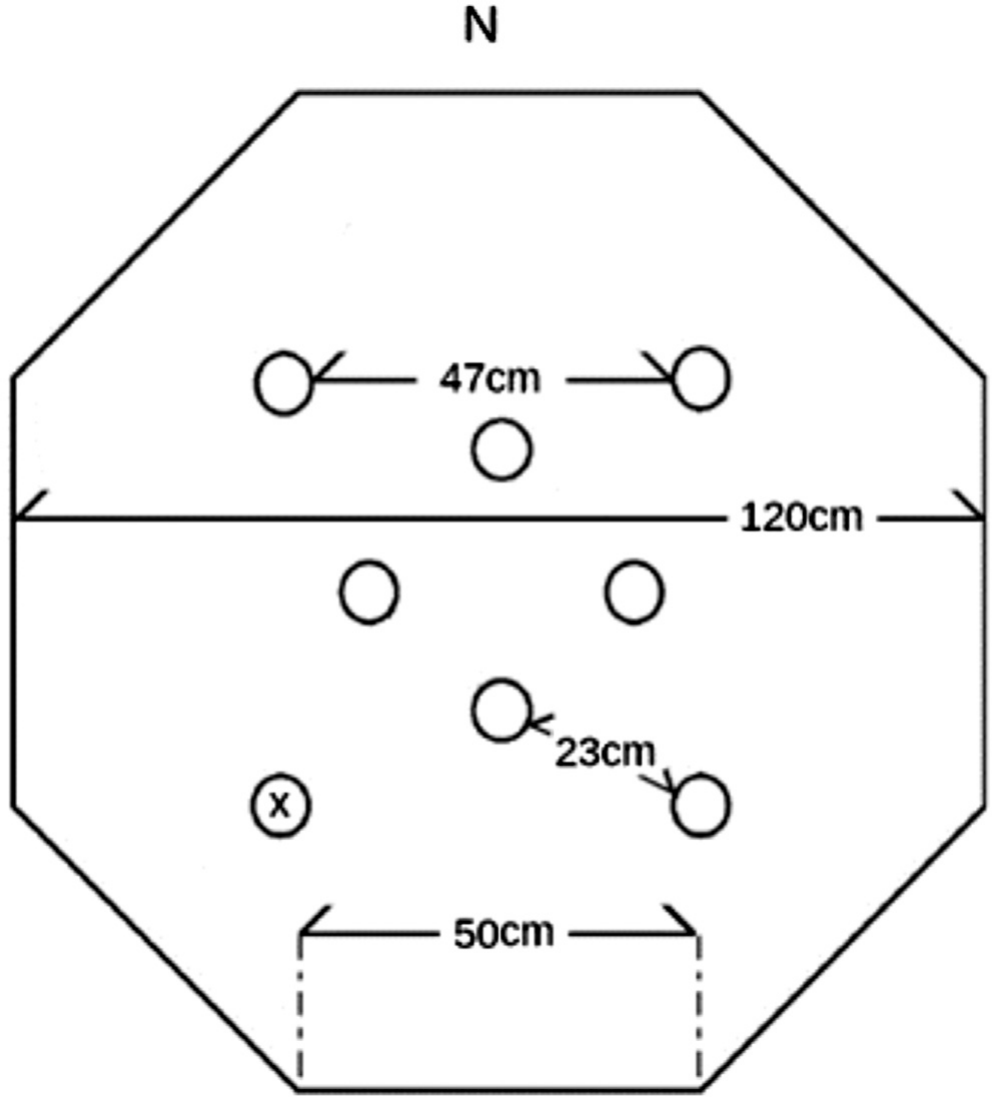
Foraging array layout Placement of cups are pictured using circles. The ‘x’ marks an example location for the baited cup (adapted from Damphousse et al., 2022).
24. 1 h prior to each step described below, remove food and transport subjects to testing room in a rack containing all subjects in individual cages. Surround cages with a curtain and leave subjects undisturbed.

***Note:*** Between subjects, wipe down the arena using 70% ethanol to eliminate scent trails.

23. Forging arena. To construct the foraging arena using standard components, we recommend using white corrugated plastic sheeting arranged into an octagon (each wall 50 cm in length, 45 cm in height). The flooring should also be constructed of the same sheeting (120 cm × 120 cm).
  a. Construct 8 unique visual cues from black poster board (ex. heart, star, circle, etc.). Four cues should be secured to walls within the maze and the other four attached to the walls of the room near the ceiling, making sure they are visible to the subject from inside the arena.
  b. Construct 8 food cups using a 3.25 oz plastic cup (height: 3.17 cm, bottom diameter: 5.4 cm, top diameter: 7.3 cm), with a 2 oz cup (height: 3 cm, bottom diameter: 4.13 cm, top diameter: 6.9 cm) with a perforated bottom nested within it (Figure 2). In the outer cup, place dried mealworms (to control for scent cues) and firmly secure the smaller cup inside. Surround inner and outer cups with tape so that worms are not visible. Attach to a piece of white corrugated plastic sheeting (8.25 cm square) to improve stability. Food cups are placed in the arena in the configuration depicted in Figure 3 and secured using double sided tape.

25. Habituation. There are 5 total days of habituation.
  a. On Days 1 and 2, habituate the quail to transport. On these days, only conduct step 24.
  b. On Days 3–5, habituate the quail to the arena. Place the subject into the center of the arena with all cups baited with one mealworm. Film behavior using an overhead webcam and record the number of mealworms eaten. Remove the subject once all worms have been consumed or after 600 s has elapsed.
26. Training trials. There are 8 total days of training with 3 training trials per subject per day. During training, only one cup is baited, and this remains consistent throughout all training trials.
  a. Place the subject into the arena at 1 of 3 locations (N, S, E) chosen at random for each trial.
  b. Record the session using overhead camera.
  c. Stop the trials either after the subject retrieves worms from the baited cup or after 300 s have elapsed. Record the latency to reach the baited cup.
  d. Return the subject to the holding cage for 1 h.
  e. Repeat a-d until all 3 trials are complete. Remember to start from 1 of the 3 start locations without repeating positions on a given testing day.
27. Probe trial. This phase is 1 trial, occurring over 1 day. During this trial, no cups are baited.
  a. Place the subject into the arena from novel direction (W) for 2 min.
  b. Record the session using an overhead camera. Record the latency to reach the target cup.

### Spontaneous object recognition

**Timing: 4 days**

The following step can be used to assess object recognition memory in Japanese quail.

28. Testing arena. To construct the testing arena using standard components, we recommend using white corrugated plastic sheeting arranged into a square (each wall 90 cm in length, 45 cm in height). The flooring should also be constructed of the same sheeting (90 cm × 90 cm) and covered in wood shavings.
  a. Cover the inside of one of the walls in black Bristol board.
  b. Select 2 different kinds of junk objects. Three copies of each object will need to be obtained so that the same object is never used twice for one bird. A variety of stimulus objects can be used. However, attempt, if possible, to satisfy the following criteria (see Figure 4 for example objects):
    i Objects must be constructed from washable materials including plastic, glass, and aluminum.
    ii Objects must be devoid of biologically relevant features such as eyes and mouths, and likenesses to food or nesting materials.
    iii Objects should range from 10 to 20 cm in height and vary in visual and tactile characteristics.

**Figure 4:**
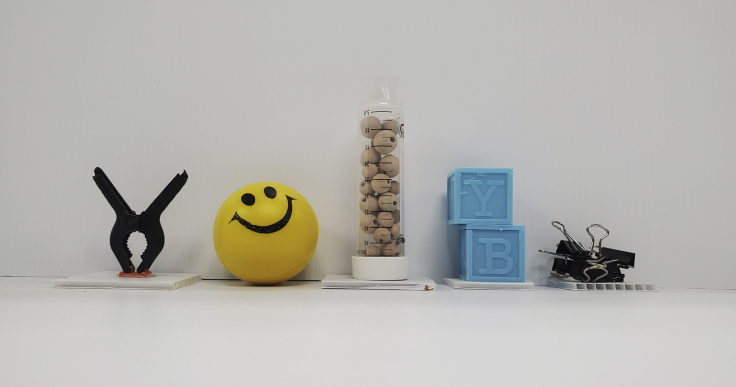
Objects used for SOR Examples of ‘junk’ objects used during SOR (from Damphousse et al., 2022, used with the permission of the authors)
29. 1 h prior to all phases described below, remove food and transport the subjects to the testing room in a rack containing all subjects in individual cages. Surround the cages with a curtain and leave the subjects undisturbed.
30. Habituation. There are 3 consecutive days of habituation to the empty arena.
  a. Place each quail in turn into the arena and allow each to explore freely for 10 min.
  b. Repeat step ‘a’ once a day for a total of 3 days.
31. Recognition Testing. Both sample and choice phases occur on the same day with a 1-min inter-trial interval (ITI; Figure 5).
  a. Sample Phase. Two identical objects are placed within the arena. Place the subject into the arena to explore freely for 5 min.
  b. Record behavior using an overhead camera.
  c. Move the subject from the arena to a holding cage for 1 min. During this time, redistribute shavings within the arena to control for scent cues.
  d. Choice Phase. Place 2 objects that the subject has yet to see within the arena. One of these objects should identical to those used during sample (familiar), while the other is novel. Place the subject into the arena to explore freely for 5 min.
  e. Record behavior using an overhead camera.
  f. Return the subject to their individual cage.

**Figure 5:**
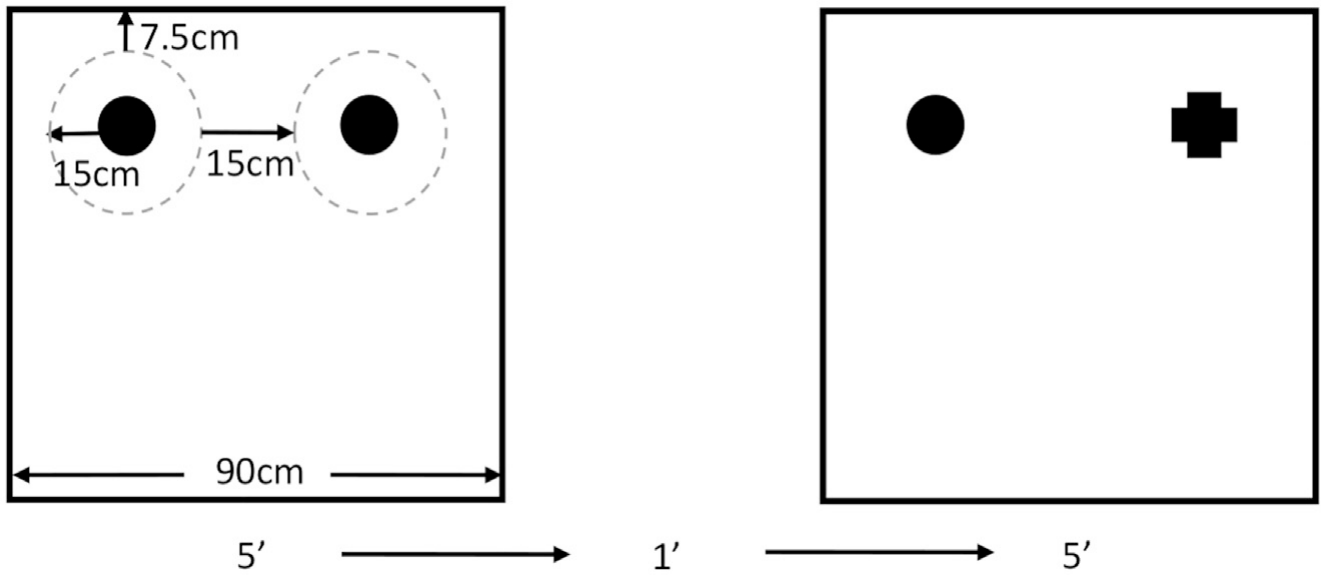
SOR Arena layout and trial timing Schematic demonstrating the placement of objects and timing of trials. The left box is an example of arena layout (with two identical objects) during the sample phase and the right depicts the choice phase in which one object identical to the sample objects is used alongside a novel object. The dashed gray circle depicted in the sample phase is to illustrate the area in which the entire body of the quail must be present for “exploration” to be occurring. This dashed circle is not physically present within the area and is instead being used for visualization of the region of interest (adapted from Damphousse et al., 2022).

### Histology

**Timing: ∼3 days**

The following step can be used to assess the extent of tissue damage caused by the Hp and APH aspiration lesions.

32. Transport subjects to a procedure room and anesthetize using isoflurane (5% isoflurane delivered in 500 mL/min oxygen).
  a. Decapitate subject and extract brain, immediately flash freezing in a container of 2-methylbutane that has been cooling within in a dry-ice and alcohol slurry.
  b. Remove brain from slurry using forceps, wrap in aluminum foil, place in airtight container (like a 50 mL centrifuge tube) and put container in a cooler of dry-ice.
  c. Once all tissue is collected, airtight containers should be placed within a resealable bag and stored at −80°C until sectioning.

**CRITICAL:** Removing the brain and working with the frozen tissue should be done quickly. If too much time elapses between brain extraction and freezing, the tissue may lose rigidity and the lesion may later appear distorted. Additionally, wrapping the tissue in foil and subsequent transport to a −80°C freezer should be done quickly to avoid thawing.

**Pause point:** When stored as described, the tissue remains viable indefinitely.

33. Sectioning. Embed tissue in mounting medium and cut sections at 30 μm thickness with a cryostat.
  a. Thaw-mount onto Superfrost Plus™ slides, air-dry slides, place in slide box wrapped in plastic wrap, place inside airtight bag and store at −80°C.
34. Collect every 6th section and stain using Nuclear fast red-aluminum sulfate.
35. View and record the placement and extent of the lesions using a brightfield microscope.

## Expected outcomes

Expected outcomes will vary according to lesion location of interest. In the study from which this protocol was adapted (Damphousse et al., 2022), lesions to Hp created deficits in the FA task but spared performance during the SOR task. Deficits on both tasks were observed following APH lesion.

## Quantification and statistical analysis

1. FA quantification and statistical analysis.
  a. For each day of training, calculate the mean latency to reach the target cup as well as the number of choices prior to reaching the baited cup. Analyze data using a repeated measures analysis of variance (ANOVA).
    ***Note:*** If comparing the effects of Hp and APH lesion on behavior, when using the repeated measures ANOVA, lesion location should be the between subject factor and training day the within subject factor.
  b. Analyze probe trials by comparing the mean proximity of each quail to the previously baited cup relative to the cup on the opposite side of the maze using a paired t-test within each group.
    ***Note:*** For Damphousse and colleagues (2022), all statistical analyses were conducted using JASP (JASP team, 2021).

2. SOR quantification and statistical analysis.
  a. The amount of time spent in exploration of each object is manually scored using the parameters listed below.
    i Exploration is defined as the bird spending time within 30 cm of an object while not preening or pecking at the surrounding walls.
    ii The entire body of the subject must be within the defined 30 cm radius and orientation of the subject toward the object is not required.
  b. Using the time spent exploring the novel (N) and familiar (F), calculate a discrimination ratio (DR) as follows: DR = (N - F) / (N + F).
    ***Note:*** DR scores range from -1 (which indicates that the bird explored the familiar object exclusively) to 1 (all exploration time was spent around the novel object). Finally, a DR of 0 would indicate an equal amount of time around both objects (consistent with random chance).
  c. Statistical Analysis. Compare mean DR for each quail across object sets using a one-way ANOVA for lesion location.
    ***Note:*** For Damphousse and colleagues (2022), all statistical analyses were conducted using JASP (JASP team, 2021) using Tukey’s HSD in all post hoc tests.

## Limitations

The current protocol has previously been used solely to examine female Japanese quail, therefore potential sex differences in performance cannot be ruled out. In addition, it is not known how well this protocol may generalize to other species of birds, particularly species better adapted to flight, as this is known to significantly influence information processing in the hippocampal formation of mammals.

This protocol is intended to provide a standardized approach for testing spatial memory and object recognition memory following lesions in Japanese quail. While this protocol contains FA and SOR procedures, there are numerous other ways to test both spatial memory and object recognition memory which are not outlined here. We are not implying that this is an exhaustive examination of spatial memory or object memory and the protocols described are instead intended to serve as a potential starting point for the study of each.

The order of the protocols described follows those in the study by Damphousse et al. (2022). While the authors cannot speak to its effectiveness, we see no reason why the order of the procedures cannot be altered to fit the experimenter’s needs. For example, FA and SOR testing may be done in the reverse order or counterbalanced, and behavioral testing may also be conducted before and after lesion depending on the desired experimental design.

## Troubleshooting

### Problem 1

Anesthetic depth too light, or the subject wakes up during surgery (steps 4 through 16).

### Potential solution

Before making surgical alterations, seek veterinarian consultation. The respiratory rates of birds are inversely related to their body weights. Because their respiratory rates are high, anesthesia can be difficult to maintain relative to mammals. The best percentage of isoflurane flow to maintain the subject at while undergoing surgery may vary between species and between subjects of varying weight. Therefore, adjust step 4 accordingly. Additionally, increasing flow rate may produce the desired effect.

### Problem 2

Quail losing weight during post-surgical period (after step 20).

### Potential solution

If quail are rapidly losing weight following surgery, your institutional veterinarian should be contacted immediately. They may suggest adding supplementary food to the subject’s diet such as dried mealworms or an egg solution (one part egg contents, one part water). This emphasizes the importance of keeping well documented post-surgical records. Thorough documentation of the subject’s condition can provide vital information for your veterinarian to develop an effective course of treatment.

### Problem 3

Infection at incision site (after step 20).

### Potential solution

Any medical intervention must first involve consultation with the institution’s animal care staff and veterinarian before beginning treatment. While infection at the surgical site is rare, this can be treated with a wash of 3% hydrogen peroxide (H_2_O_2_). Your vet may also consider extending injectable antibiotic treatment or may recommend a topical antibiotic ointment.

### Problem 4

Sign of infection in brain tissue revealed during histology (steps 33 through 35).

### Potential solution

If during histology, the brain tissue at the lesion site appears to be a color differing from that of healthy pink tissue (ex. yellow, brown, or green), the subject’s data should be excluded from the study. Brain infections, usually caused by a lack of sterile procedures during surgery, can cause behavioral changes during testing. In this case, discriminating between behavioral changes attributed to the lesion and to the infection is impossible. The subject’s data can result in inaccurate and misleading conclusions and must therefore be excluded.

### Problem 5

Subject not moving when introduced to FA or SOR arena (steps 23 through 31).

### Potential solution

Japanese quail require a great deal of habituation and are easily stressed relative to common mammalian research models. This may also be true of other birds. Ensure that habituation steps are being followed properly and that the testing environment is quiet and free of startling noises. In addition to environmental habituation, quail should also be habituated to experimenters and the number of experimenters should be kept to a minimum.

## Resource availability

### Lead contact

Further information and requests for resources and reagents should be directed to and will be fulfilled by the lead contact, Chelsey C Damphousse (damp7000@mylaurier.ca).

### Materials availability

There are no newly generated materials associated with this protocol.

## Data Availability

No new data or code was generated for this protocol.

## Supplemental information

Data S1. Example surgical and post-surgical monitoring log, related to step 21
