# Supplementary material for: Protocol for inducing hippocampal formation lesions and associated behavioral testing in Japanese quail
Source: STAR Protoc. 2022 Jul 18;3(3):101553. doi: 10.1016/j.xpro.2022.101553 (PMC9304673; doi:10.1016/j.xpro.2022.101553)
Supplement: Data S1. Example surgical and post-surgical monitoring log, related to step 21 [file mmc1.pdf]

Data S1. Example surgical and post-surgical monitoring log. Related to step 21.

Surgical and Post-Surgical Monitoring Log (Avian)

Researcher: ____________ **Contact:** _________ **Principal Investigator:** _________ **Contact:** _________
Animal ID (Species-Identifier): _____________ **Protocol #:** ____________________
Start Weight: ______________

Surgery Day			
Date	Event	Notes	
	Surgery Day	Start Time:	
	Type:	End Time:	
		Analgestic:	
		Dosage:	Given:
		Anti-Biotic:	
		Dosage:	Given:
		Additional Info:	

Post-Surgical			
Date	Event	Notes	
	Post-Op Day 1	Weight:	Inj. time:
		Analgestic (mL):	Anti-Bio (mL):
	Post-Op Day 2	Weight:	Inj. time:
		Analgestic (mL):	Anti-Bio (mL):
	Post-Op Day 3	Weight:	Inj. time:
		Anti-Bio (mL):	
	Post-Op Day 4	Weight:	inj. time:
		Anti-Bio (mL):	
	Post-Op Day 5	Weight:	inj. time:
		Anti-Bio (mL):	
	Post-Op Day 6	Weight:	inj. time:
		Anti-Bio (mL):	
	Post-Op Day 7	Weight:	
		*remove sutures	

BAR= Bright, Alert, Responsive
